# Structure-from-Motion Approach for Characterization of Bioerosion Patterns Using UAV Imagery

**DOI:** 10.3390/s150203593

**Published:** 2015-02-04

**Authors:** Sibila A. Genchi, Alejandro J. Vitale, Gerardo M. E. Perillo, Claudio A. Delrieux

**Affiliations:** 1 Instituto Argentino de Oceanografía, CONICET, CC 804, B8000FWB Bahía Blanca, Argentina; E-Mail: gmeperillo@criba.edu.ar; 2 Departamento de Geografía y Turismo, Universidad Nacional del Sur, 8000 Bahía Blanca, Argentina; 3 Departamento de Ingeniería Eléctrica y de Computadoras, Universidad Nacional del Sur, 8000 Bahía Blanca, Argentina; E-Mail: cad@uns.edu.ar; 4 Departamento de Geología, Universidad Nacional del Sur, 8000 Bahía Blanca, Argentina

**Keywords:** Structure-from-Motion Approach, unmanned aerial vehicle, 3D point cloud, topography, bioerosion, burrowing parrot

## Abstract

The aim of this work is to evaluate the applicability of the 3D model obtained through Structure-from-Motion (SFM) from unmanned aerial vehicle (UAV) imagery, in order to characterize bioerosion patterns (*i.e.*, cavities for roosting and nesting) caused by burrowing parrots on a cliff in Bahía Blanca, Argentina. The combined use of SFM-UAV technology was successfully applied for the 3D point cloud model reconstruction. The local point density, obtained by means of a sphere of radius equal to 0.5 m, reached a mean value of 9749, allowing to build a high-resolution model (0.013 m) for resolving fine spatial details in topography. To test the model, we compared it with another point cloud dataset which was created using a low cost do-it-yourself terrestrial laser scanner; the results showed that our georeferenced model had a good accuracy. In addition, an innovative method for the detection of the bioerosion features was implemented, through the processing of data provided by SFM like color and spatial coordinates (particularly the *y* coordinate). From the 3D model, we also derived topographic calculations such as slope angle and surface roughness, to get associations between the surface topography and bioerosion features.

## 1. Introduction

The new generation of remote sensing and computing technologies has changed the way in which topographic data is acquired and treated. In this framework, photogrammetry (terrestrial and aerial), despite its long history, has only recently emerged as a promising competitor to laser scanning systems [1,2], mainly due to the fact that the former has been integrated into advanced computer vision solutions. The development of a powerful approach called Structure-from-Motion (SFM), which combines well-established photogrammetric principles (basically, image matching and bundle adjustment) with modern computational methods [3], has played a key role in these advances. This trend has been confirmed in many studies involving topographic surveys on different environments such as architectural [4–6], forest [7–9] and, more recently, geomorphologic environments [10–13]. The SFM approach estimates simultaneously the scene structure and camera motion from multiple images [14]. Since the last years, numerous SFM software packages are available, allowing one to obtain fully automated high-resolution topographic reconstructions from unstructured and unconstrained image collections using consumer-grade cameras.

Besides the aforementioned advances in the field of digital photogrammetry, unmanned aerial vehicles (UAV) have also been introduced as a promising technology, providing new opportunities mainly for the study of remote or inaccessible areas [15]. This equipment poses a wide range of survey configurations, allowing to ensure the optimal image capture (e.g., overlap), which becomes essential for the requirements of the algorithmic developments in computer vision. At the present, the SFM approach is of great interest in the processing of images acquired by UAV; missing or incorrect data (*i.e.*, on-board camera parameters) can be solved by using SFM, since it does not require any parameter for its implementation. Tonkin *et al.*'s work [16] makes reference to a new methodological frontier for topographic data acquisition based on UAV-SFM, of great interest for scientists, especially among geomorphologists.

**Figure 1.**
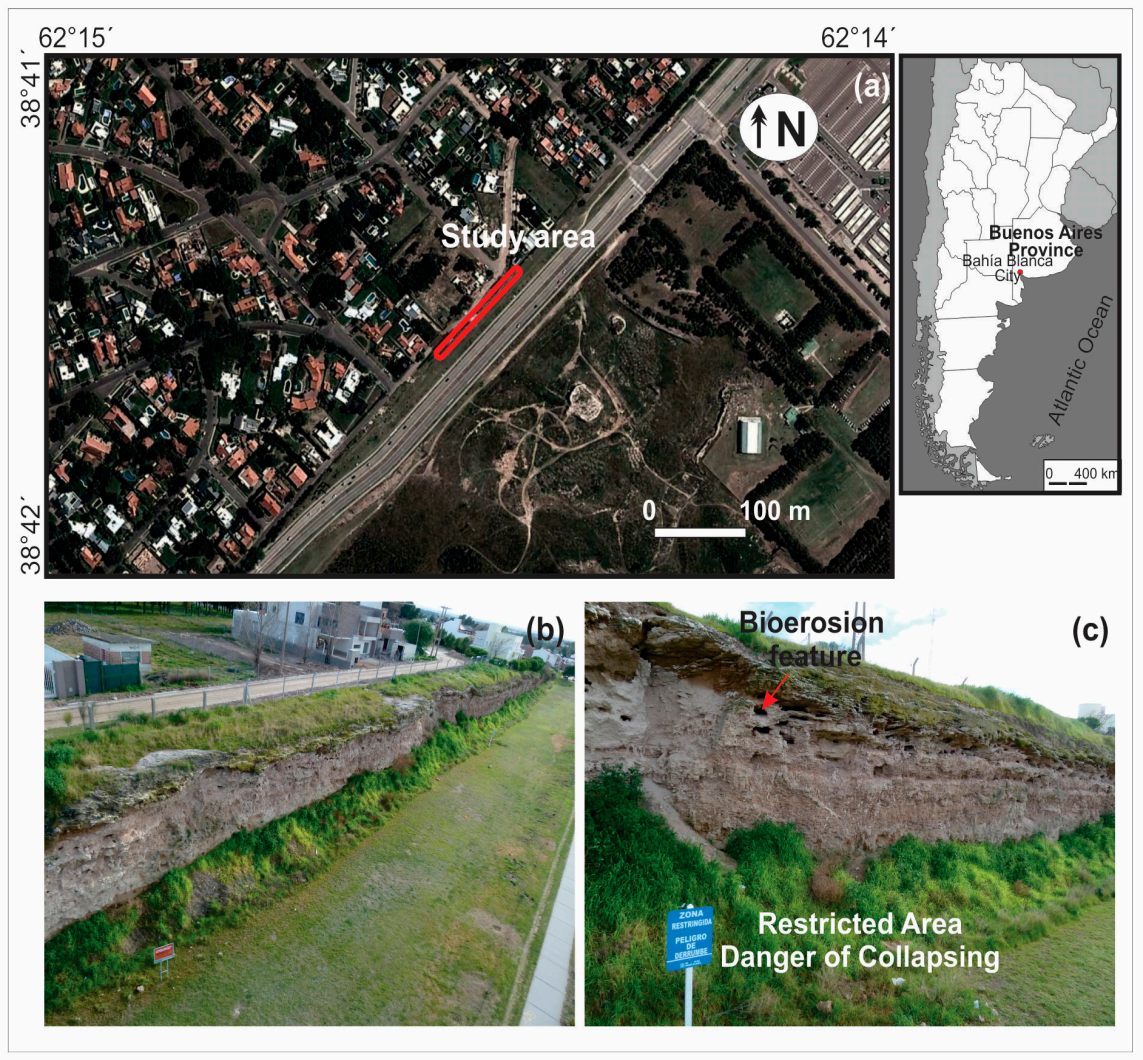
(**a**) Location map of the study area; (**b**,**c**) Aerial views of the bioeroded cliffy landform.

Biogeomorphology, which considers the interrelations between biological and geomorphological processes [17], is particularly well-suited for the understanding of erodible surfaces, such as is the case of soft rocks. Here there emerges the concept of bioerosion, which was introduced to the literature by Neumann [18] in 1966 as the removal of consolidated substrate by the direct action of organisms. Since then, many studies of bioerosion were focused on the activity of one or more organisms, mainly over marine carbonate environments in tropical-subtropical [17–20] and temperate-cold [21,22] regions, which aimed to assess bioerosion rates and, in less measure, to quantify spatial patterns. The main aim of this work is to evaluate the applicability of the 3D model obtained through SFM from UAV imagery, in order to characterize bioerosion patterns (*i.e.*, cavities for roosting and nesting) caused by urban burrowing parrots on a vertical surface (cliff) located in Bahía Blanca, Argentina (Figure 1).

## 2. Study Area

The study area, called “Barrancas de Sarmiento”, is located very close to buildings on the north side of the city of Bahía Blanca, Buenos Aires Province, Argentina (Figure 1a). The climate is temperate and sub-humid, with warm summers and cold winters. This land strip constitutes a soft-rock (Pampean sediments) cliff system of ∼8 m height, whose origin is probably related to a marine transgression [23]. This vertical surface is physiographically adequate for roosting and nesting bird species, as is the case of the burrowing parrot (*Cyanoliseus patagonus*). Consequently, in the cliff face, a large number of cavities (hereinafter called bioerosion features—BF) can be observed as a result of bioerosion processes, signifying danger of collapsing in some sites (Figure 1b,c). The study area is the only existing urban colony, being regularly visited by international bird watchers.

## 3. Methodology

### 3.1. Data Collection

**Figure 2.**
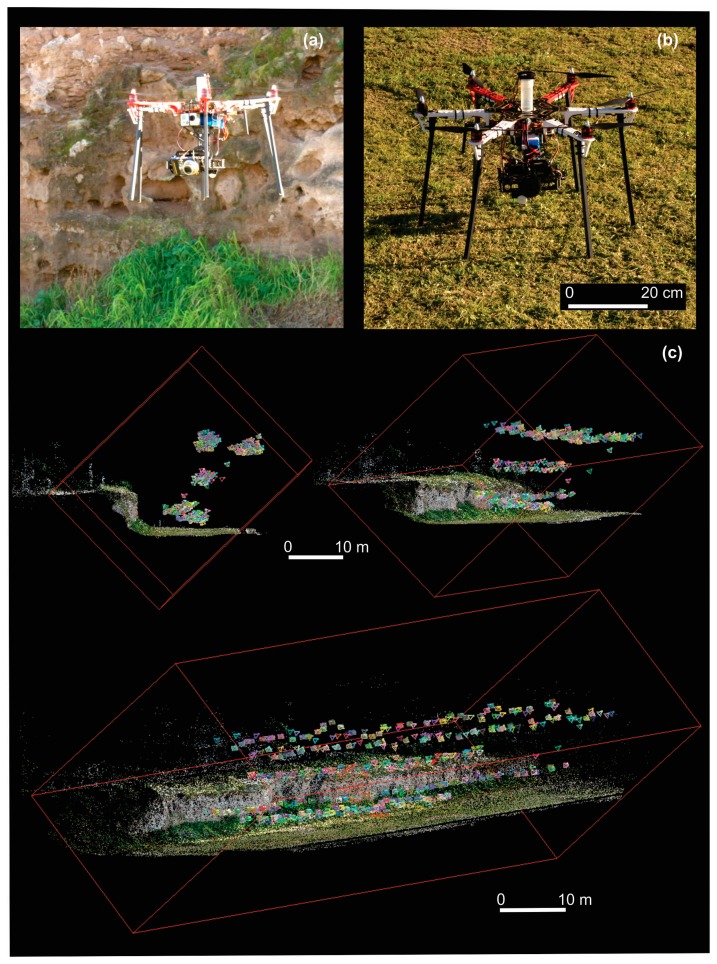
(**a**,**b**) UAV hexacopter; (**c**) Three different views of all the camera (and photos) positions showing the UAV track along the vertical surface; also, the scattered point cloud (323,847 points) built from the matching can be seen.

The 3D model was achieved using images taken with a consumer-grade camera (Lumix DMC-TZ10, 12 MP, Panasonic, Osaka, Japan) mounted on a UAV. The flight vehicle is a hexacopter (DJI F550 air frame, DJI, Shenzhen, China) commanded by an open-source ArduPilot Mega 2.6 with a generic 2D gimbal (Figure 2a,b). The UAV includes an on-board GPS/IMU, logging at 5 Hz. Its total weight is about 3 kg. The estimated price, including camera and hexacopter (complete kit), is around US$1250.

To ensure high degree of overlap, flight speed was set at 1 m s^−1^ and the images were taken at 3 m intervals, resulting in nearly 400 images. The UAV followed a predefined flight path, covering a set of flight lines (five in total) which are parallel to the vertical surface (Figure 2c). Each flight line was duplicated, acquiring two views on the same surface, with an angle of approximately 25° (Figure 2c). The total flight time took about 30 min. The field work was conducted in the early morning, when the illumination of the whole area was sufficiently homogeneous.

### 3.2. 3D Model Reconstruction

After carrying out the selection of images to be analyzed, the first step was to identify and match features automatically in consecutive images through an object recognition system called scale invariant feature transform, using the open-source software package VisualSFM [24]. The approach transforms an image into a large set of local feature vectors. The features are invariant to the image scaling and rotation, but they are partially invariant to changes in illumination conditions and 3D camera viewpoint [25]. Within this process, all unstable points were filtered. A second step was to estimate both intrinsic (geometric and optical characteristics) and extrinsic (3D position and orientation of the camera frame in a relative coordinate system) camera parameters by determining optimal camera positions using the bundle adjustment algorithm into the VisualSFM software. A sparse point cloud is built, representing the most important features (Figure 2c).

In order to generate a dense point cloud, Clustering Views for Multi-view Stereo (CMVS) and Patch-based Multi-view Stereo (PMVS) algorithms were applied using the VisualSFM software. The software provides a convenient menu interface to run external tools like the aforementioned algorithms. The previously determined camera positions were used as input. CMVS decomposes input images into clusters (sub-models) and PMVS reconstructs 3D data for each cluster [26].

### 3.3. Georeferencing

The resulting dense point cloud (11,364,917 points) is generated in an arbitrary (image) coordinate system, which must be transformed into an absolute coordinate system. To achieve this transformation, firstly, eight well-distributed anthropogenic benchmarks along the cliff (four posts in the upper part; three safety cones and one sign board in the lower part) were considered to aid the location in the point cloud. The 3D position of these points was performed using a Sokkia Radian IS real-time kinematic differential Global Positioning System (DGPS-RTK) unit (Sokkia, Mississauga, Canada) in order to obtain high accuracy (horizontal accuracy = 10 mm, vertical accuracy = 20 mm). The location is given in UTM coordinate system (zone 20S) that then was converted into manipulable data. Finally, georeferencing process of the 3D point cloud was carried out in VisualSFM software. This software allows to combine all the resultant sub-models into a single, larger-scale model and, once georeferenced, permits to save the model in PLY format (Stanford Triangle Format).

### 3.4. Testing the Model

With the aim of testing the model, we collected 3D coordinates for a large number of points over a portion of the vertical surface by using a low cost do-it-yourself (DIY) terrestrial laser scanning (TLS). Therefore, both SFM photogrammetry and DIY-TLS point cloud datasets were finely aligned and then compared in terms of absolute distance by means of the open-source software CloudCompare [27].

The DIY-TLS (previously tested) consists of a commercial laser range finder (model UT390B, Uni-T, Shanghai, China; accuracy: ±2 mm) that rotates around two axes (*x*, *y*) using stepper motors; the equipment is mounted on a tripod. The sampling rate is 1 point every 1.1 s. A program was developed to control scanning motion and laser pulses using the Arduino open-source electronics prototyping platform (Mega 2560, Arduino, Torino, Italy).

Also, a radio telemetry system (3DR radiotelemetry Kit-915 MHz, 3DR, San Diego, CA, USA) with a range up to 500 m was used. Data extraction from the DIY-TLS is based on time-of-flight of the laser pulse to travel from its source to the target object and back; therefore, it computes the distance based on the travel speed of the pulse [28]. Coordinates of each point are acquired in a polar coordinate system (ρ, ϑ, φ). Subsequently, these coordinates were transformed into the Cartesian system (*x*, *y*, *z*) and corrected based on known points of reference (DGPS-RTK).

### 3.5. Calculations Derived from the Model

Three properties (for each vertex) were stored in PLY file format: spatial coordinates (*x*, *y*, *z*), normal (*nx*, *ny*, *nz*) and color (*RGB*). To carry out post-run analysis, the georeferenced model (PLY format) was saved in ASCII format using the CloudCompare software. Then, all the header field values in the text file (“.txt”) were removed. The analysis was run under the GNU Octave software [29]. In general terms, two sets of calculations were derived from the model: one corresponding to the topographic properties of the vertical surface, and the other corresponding to the detection of the BF. Finally, associations between the results were carried out using bivariate statistical analysis.

#### 3.5.1. Processing Step of Model Data

In a first processing step on the georeferenced point cloud (ASCII format), the work area (study area) to be processed was delimited. This area comprises the cliff face excluding vegetation entirely (0 < *x* < 120, 15 < *y* < 25, 4 < *z* < 8 m). The following step consisted of sorting the entire point cloud data to obtain a 2D matrix with nine parameters (*i.e.*, 3D spatial, normal and color information). The latter is a user-defined matrix (*m*x*n*) according to the amount of points and the real length. Thus, the spatial resolution (cell size) for the matrix of 10,610 × 354 was 0.0113 m.

#### 3.5.2. Calculations Based on Topographic Properties of the Vertical Surface

The slope angle (θ) is calculated locally over a 3 × 3 window by the maximum rate of change in elevation between each cell and its neighbors as follows (modified from Burrough and McDonnell [30]):

(1) $$\theta =\arctan\left(\sqrt{{\left[\frac{dy}{dx}\right]}^{2\;}+\;\;{\left[\frac{dy}{dz}\right]}^{2\;}}\right)\frac{180}{\pi }$$

where $\frac{dy}{dx}$ and $\frac{dy}{dz}$ are the rates of change in the horizontal and vertical directions from the centre cell, respectively.

Local topographic roughness of the work area was calculated using circular statistic and Laplacian operator methods. With respect to the former, we applied the circular standard deviation from normal data. The resultant length or the sum of normal unit vectors (*R*) can be computed in 3D as [31]:

(2) $$R={\left({\left(\sum^{\;}x_{i}\right)}^{2}+{\left(\sum^{\;}y_{i}\right)}^{2}+{\left(\sum^{\;}z_{i}\right)}^{2}\right)}^{½}$$

The circular average (*RC_avg_*) is obtained as follows [32]:

(3) $$RC_{\mathrm{avg}}=\frac{R}{n}$$

where *n* is the number of cells in a given window. From Equation (3), we can calculate the circular standard deviation (*RC_sd_*) which is given by the following expression [31]:

(4) $$RC_{sd}={\left(-2\log\left(RC_{\mathrm{avg}}\right)\right)}^{½}$$

The Laplacian operator used here calculates the two-dimensional topographic curvature employing a standard five-point central difference formula. Thus, the calculation is performed in the following way (modified from McKean and Roering [32]):

(5) $$\left|\nabla^{2}y\left(x,z\right)\right|=\mathrm{abs}\left(\left(\frac{y_{E\;}-2y+y_{W}}{\Delta x^{2}}\right)+\left(\frac{y_{N\;}-2y+y_{S}}{\Delta z^{2}}\right)\right)$$

where *y_E_*, *y_W_*, *y_N_* and *y_s_* are the cardinal directions in the window.

#### 3.5.3. Calculations Based on Bioerosion Features Detection

BF were detected from color and spatial coordinates properties jointly. Firstly, adjustments were made to each of the properties. A conversion of color information from *RGB* to luminance was made. The luminance (*L*) can be calculated from linear *RGB* components as follows:

(6) L=0.2126 R + 0.7152 G + 0.0722B

The spatial coordinate *y* (*i.e.*, 15 < *y* < 25) was normalized to 1.

After some tests, we choose a moving window of 7 × 7 cells (*i.e.*, ∼8 cm wide) to implement the calculations. We computed an average value of luminance (*L_avg_*) and *y* spatial coordinate (*Y_avg_*) that is ascribed to a single cell at the centre of the window. Subsequently, as a result of comparing this value with those 10 previously obtained values (*L_10avg_* and *Y_10avg_*), the resultant value in the cell *ij* will acquire a binary value (0 or 1) according to the following:
 **If** (*L_avg_* < 3 dB of *L_10avg_*) **and** (*Y_avg_* < *Y_10avg_*) **then**   cell *ij* = 1, whenever both conditions are satisfied **else**   cell *ij* = 0, when at least one condition is not satisfied.

Once the BF were detected, the next step was to calculate the area (*A*) and perimeter (*P*) from the construction of polygons by geometrical computation. For this, we run the matrix elements through a systematic method that uses morphological operators within a square window (7 × 7 cells). Then, we calculated a shape factor called circularity index (*C*) by the following expression:

(7) $$C=\frac{4\;\pi \;A}{P^{2}}$$

A circularity value of 1 represents a perfect circle, while decreasing values correspond to more irregular shapes. For the further analysis, we considered BF whose area is greater or equal to 50 cm^2^.

## 4. Results and Discussion

### 4.1. 3D Point Cloud Model

**Figure 3.**
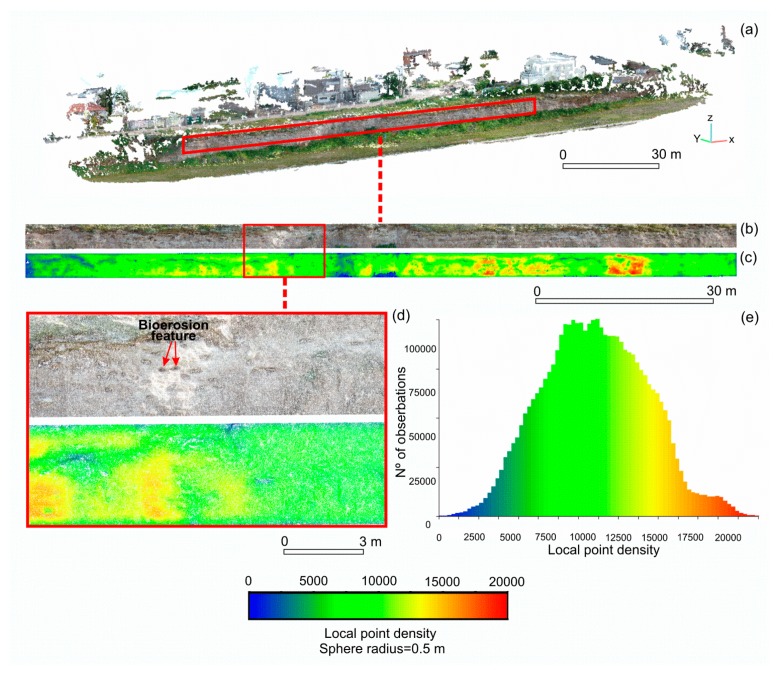
(**a**) Dense point cloud of the total area (11,364,917 points) indicating the study area; (**b**) Dense point cloud of the study area (3,714,511 points); (**c**) Map of local point density of the point cloud obtained using a sphere of radius equal to 0.5 m; (**d**) Zoom of the area where there is a significant number of bioerosion features; (**e**) Histogram of the frequency of occurrence of local point density of the study area.

A perspective view of the dense point cloud model covering the total area is presented in Figure 3a. As was mentioned before, the sparse point cloud comprised 323,847 points, while the dense point cloud produced 11,364,917 points. A map of local point density of the 3D model corresponding to the study area (*i.e.*, 3,714,511 points) is shown in Figure 3c. The map was created from a sphere of radius 0.5 m by using the CloudCompare software. Clearly, the point density is more concentrated over the most prominent portion of the vertical surface, due to the fact that the point density is inversely proportional to the distance from the equipment. In this latter case, the values of density were greater than 12,500. Low values of point density (from 1000 to 2500) in specific portions of the study area are attributed to the presence of complex topography, which might cause an inefficient data capture (Figure 3c). In general terms, although there is a slightly lower local point density inside of the BF compared to the rest of the surface (Figure 3d), it is noticeable that these points tend to lose detail in *y* spatial coordinate from a certain depth.

Also, a histogram of the local point density is presented in Figure 3e. It showed a normal distribution, with a mean of 9749 and a standard deviation of ±3237. The local point density ranged from 5 to 20,015 points. Very low values of density at the lower end is due to occasional presence of vegetation cover.

### 4.2. Test of the Model

**Figure 4.**
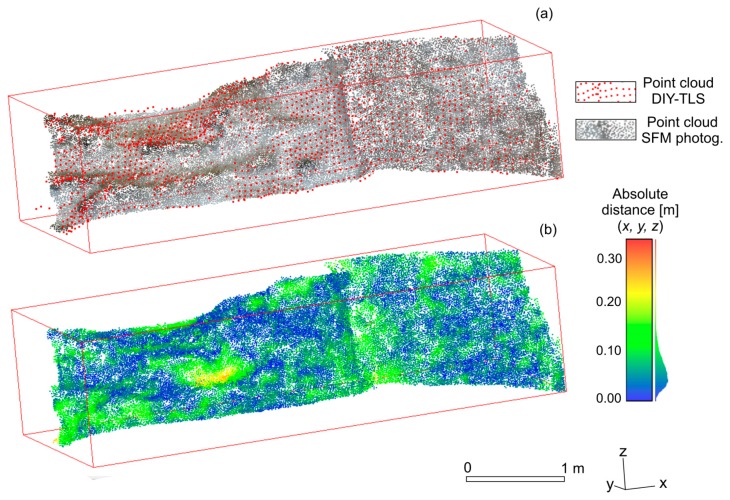
(**a**) View of the SFM photogrammetry and DIY-TLS point clouds on a portion of the study area. (**b**) Absolute distance between the two point cloud datasets, considering *x*, *y*, *z* spatial coordinates.

The test of the model was carried out on a surface of about 6.7 m^2^ containing a complex topography. A comparison of the two georeferenced point cloud datasets, that is, the SFM photogrammetry model (50,944 points) and the DIY-TLS (1761 points), was made (Figure 4a). The comparison consisted of measuring the absolute distance between each point in the compared dataset with its closest point (*i.e.*, nearest neighbor distance). In general terms, a good accuracy between the two datasets is noticeable (Figure 4b). The absolute distance variability appears not to have a clear pattern in the tested surface.

The Table 1 contains the statistical calculations of the absolute distance between the two dataset for the three spatial coordinates. Results concerning *x*, *y*, *z* coordinates jointly, indicated a mean distance of ∼0.07 m with a standard deviation of ±0.035 m. The *y* coordinate presented the greatest mean distance, with a value of 0.04 m. The remaining two coordinates showed similar values of the order of 0.03 m. It must be taken into consideration that the mean absolute (three-dimensional) distance is within the limits of the root-mean square error (0.19 m) involved in the georeferencing process of the whole SFM model.

### 4.3. Topographic Properties of the Vertical Surface

**Figure 5.**
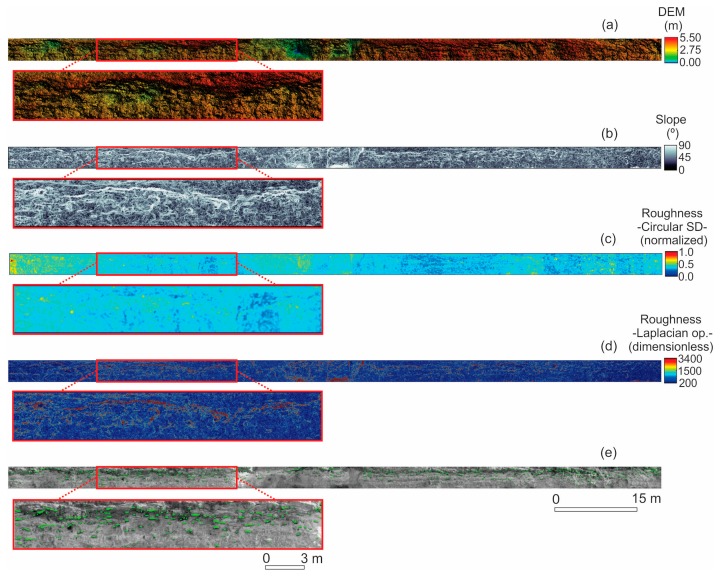
Topographic properties and bioerosion features of the study area. (**a**) Digital elevation model (DEM); (**b**) Slope angle; (**c**) Surface roughness based on circular standard deviation; (**d**) Surface roughness based on Laplacian operator. (**e**) Bioerosion features (green polygons) superimposed over the dense point cloud. Note: The DEM was created by using triangulation with linear interpolation gridding method (spacing = 0.02 m).

As a result of the achieved level of density, it was possible to obtain a high resolution model (0.013 m) for resolving fine spatial details in topography. Figure 5a–d shows all the topographic properties that were considered in this study. Calculations of slope and surface roughness properties give a local measure, independent of small-scale topographic variability, having the potential to differentiate individual surface properties to which it is most sensitive. Our results show that both local slope and surface roughness (e.g., Laplacian operator method) properties, which ranged from 0.1° to 89.3° and from 198 to 3364, respectively (Figure 5b,d), were able to demonstrate topographic patterns visually consistent with the real environment at a high level of detail.

The mean slope angle of the study area is 47°; the highest values were found in several parts of the cliff, but more significantly in the top portion (Figure 5b). In the case of surface roughness, whichever method is used, it also increases near the top of the cliff (Figure 5c,d). The roughness based on the Laplacian operator method showed a mean variability of 22%. According to the map of roughness (Figure 5d), warm colors denotes high variability, showing convex and/or concave curvature of the surface. An important pattern to note is, for example, the presence of a narrow convex-concave surface over the upper portion of the cliff.

### 4.4. Bioerosion Pattern Characterization

As a result of processing different kinds of information provided through the use of SFM approach, it was possible to detect BF, and then to characterize their geometry. In this work, an innovative method for the detection of the BF was implemented, which is given by the addition of color data to spatial coordinates (particularly the *y* coordinate). A total of 858 BF were detected in the study area (*i.e.*, vertical surface = 480 m^2^) (Figure 5e). These features comprise a bioeroded surface of the order of 5.7%, and a frequency of occurrence of 1.8 cases per square meter. However, the BF are concentrated close to the top of the cliff, at a mean height of about 6.5 m above the ground (Figure 5e).

**Figure 6.**
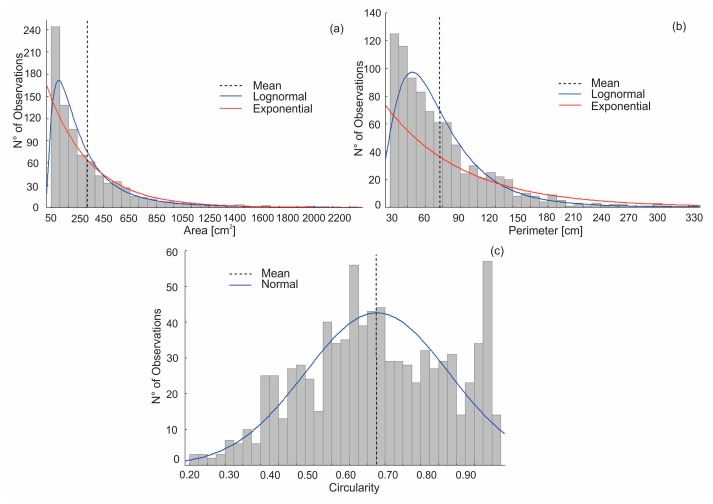
Frequency histograms of the bioerosion features variables. (**a**) Area; (**b**) Perimeter; (**c**) Circularity.

The mean area of the BF is 320 cm^2^ with a similar value of standard deviation, which is indicating a great variability in their size (Figure 6a). The frequency distribution of this variable follows a lognormal distribution as is shown in Figure 6a. There is a predominance of BF ranging from 50 to 200 cm^2^. Regarding their perimeter, it reached a mean value of 76 cm with a relatively high standard deviation (±47 cm). The frequency distribution of the perimeter also follows a lognormal distribution (Figure 6b). Circularity index showed a mean value of 0.67, which indicates BF shapes tending to be more irregular or elongated.

The standard deviation of this index was low (±0.18), implying that the variability was insignificant. The frequency distribution of the circularity index follows a normal curve (Figure 6c); also, it can be seen that the histogram shows a second and less significant peak close to values of 0.9.

**Figure 7.**
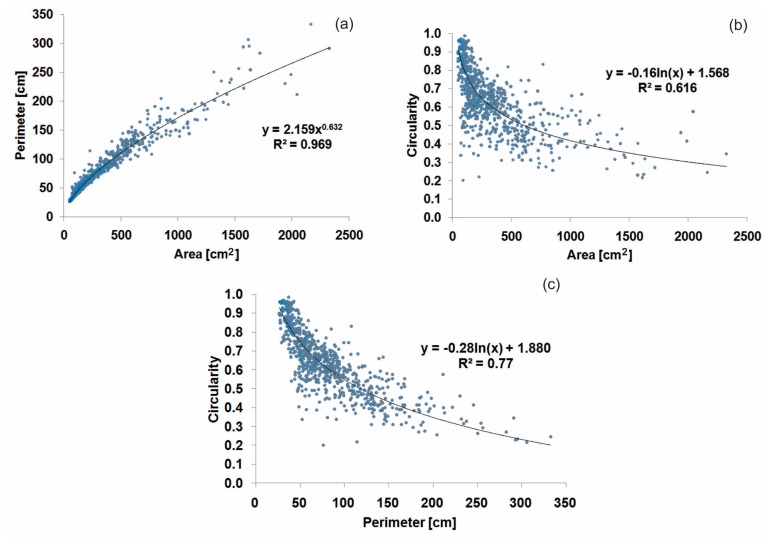
Correlations between the bioerosion features variables. (**a**) Area and perimeter; (**b**) Area and circularity; (**c**) Perimeter and circularity.

There is a high positive (potential) correlation (R^2^ = 0.97) between area and perimeter (Figure 7a), demonstrating that the variables perform equivalently, as is expected. Plotting the circularity index against either area or perimeter, it shows a negative slope, that is, decreasing circularity with increasing area or perimeter (Figure 7b,c). A relatively high (logarithmic) correlation of 0.62 and 0.77 was found between circularity and both area and perimeter, respectively; however, certain degree of correlation is unavoidable since the same data is used in both variables.

**Figure 8.**
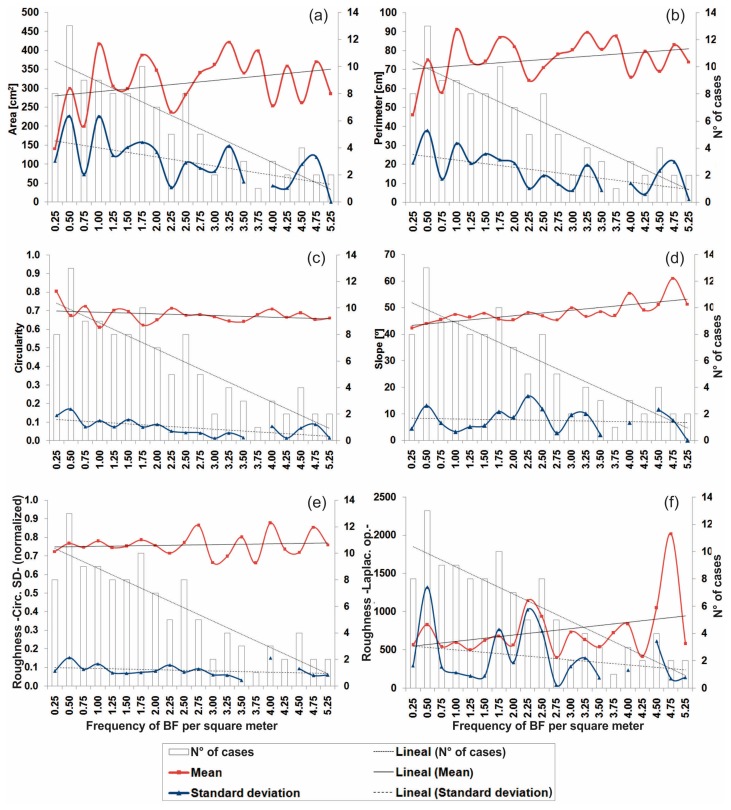
Trend plots of several variables of bioerosion features and surface topography against the frequency of occurrence of bioerosion features. (**a**) Area; (**b**) Perimeter; (**c**) Circularity; (**d**) Slope angle; (**e**) Surface roughness based on circular standard deviation; (**f**) Surface roughness based on Laplacian operator.

The study area was partitioned into 125 subareas based on equal sized cells of 4.8 × 0.8 m, in order to examine trends and interrelationships between BF and surface topography. According to the plots of Figure 8, we could explain the behaviour of the geometric (BF) and topographic (vertical surface) properties against the frequency of BF. It was possible to observe the predominance of subareas with low and, to a lesser extent, middle frequency of the BF per square meter. In the case of mean values of both area and perimeter, a clear trend to increase with an increase in the frequency of BF was observed, indicating that the largest BF are situated closer to each other (Figure 8a,b). In contrast, the standard deviation values of area and perimeter show a trend toward decrease with an increase in the frequency of BF, implying a decrease in the variability of the dimension according as they are more concentrated. The mean and standard deviation of the circularity index also presents a mild decrease with increasing frequency of BF (Figure 8c).

When increasing the frequency of BF, an increase of the mean slope angle values of the vertical surface is observed (Figure 8d). The latter indicates that the largest and most concentrated BP tend to be situated on steeper surfaces, ranging from 50° to 60° (mean values). Figure 8e,f show the surface roughness property calculated by using circular standard deviation and Laplacian operator methods, respectively. Regarding the former, there is no defined trend. However, when we use the Laplacian operator method, an apparent trend can be observed, indicating that the mean roughness (convex and/or concave curvature) increase with an increase in the frequency of BF as is expected.

### 4.5. Applications and Limitations of SFM-UAV

As proven in several studies [10,12,13] and in this work, the topography generated by SFM photogrammetry offers similar performance in resolution and precision to that of alternative approaches (e.g., TLS, aerial laser scanning (ALS), or real-time kinematic GPS) but with the advantage that it is much cheaper and easier to implement. In addition, UAV technology is fully suitable for SFM photogrammetry approach, due to its wide range of operational possibilities (e.g., position and speed control of flight) for capturing photos. Although the use of SFM-UAV technology implies costs of equipment (in this case, ∼US$1,250) and sometimes personnel to make the assessment, it is much cheaper than, for example, TLS or ALS, usually by at least one order of magnitude. These advantages are of great importance in multitemporal studies, in order to evaluate the spatial dynamics of a given surface.

Unlike laser scanning systems such as TLS/ALS, SFM photogrammetry provides at the same time 3D spatial (*x*, *y*, *z*) normal (*nx*, *ny*, *nz*) and color (*RGB*) datasets. As a result of this large amount of data, SFM photogrammetry has become more demanding regarding to post-processing tasks. Particularly, the availability of color data represents a clear advantage in terms of practicability, since by means of alternative methodologies this information is only possible to obtain by adding a digital camera mounted independently of the measurement device [33–35]. In this work, the combined use of both 3D spatial and color datasets have contributed to the determination of the BF.

Despite the aforementioned advantages, there are still limitations in the field of the photogrammetry referred to the sunlight exposure over topographically complex surfaces (e.g., cavities, depressions, *etc.*), which might be well resolved by laser scanning systems. This limitation causes pixel values near zero, difficulting the process of feature matching. In the current study, when considering the *y* spatial coordinate, the point cloud well inside the BF begins to generate erroneous data at a given depth where the sunlight never penetrates.

## 5. Conclusions

In this study, SFM-UAV technology was successfully applied in the topographic reconstruction of a large vertical surface, thereby allowing to achieve the proposed aim of characterization of the bioerosion patterns and BF properties. A comparative (*i.e.*, absolute distance) test carried out between two point cloud datasets showed that our georeferenced 3D point cloud model had a good accuracy, with a mean distance of 0.07 m. The local point density, using a sphere of radius equal to 0.5 m, indicated a mean of 9749 and a standard deviation of ±3237. This allowed to build a high-resolution model (0.013 m) for resolving fine spatial details in topography. From the 3D model, we derived topographic calculations such as slope angle and surface roughness with enough accuracy, in order to get associations between the surface topography and BF.

As a result of processing different data provided by SFM approach, that is, color and spatial coordinates (particularly the *y* coordinate) data, it was possible to detect 858 BF and to characterize their geometry. There is a predominance of BF whose area and perimeter range from 50 to 200 cm^2^ and from 30 to 60 cm, respectively, suggesting elongated shapes. The trends indicated that the largest BF are situated closer to each other, at a mean height of about 6.5 m above the ground. An apparent trend can be observed, indicating that both the slope and the mean roughness (convex and/or concave curvature) increase with an increase in the frequency of BF as is expected. From this information, we conclude that parrot population is distributed much closer to the upper portion of the cliff, where they may be less disturbed by human practices.

Finally, we could conclude that the SFM-UAV resulted in a effective alternative in terms of resolution, precision, cost and practicability. Another key aspect is the use of a non-invasive technology, particularly when considering that the study area presents roosting and nesting sites of burrowing parrots. It should be added that the use of roost/nest counting (*i.e.*, BF in the current work) proves an useful indirect method in attempting to estimate parrot population size, which became vital for their conservation and management. Likewise, the characterization of spatial patterns of bioerosion constitute an essential step in the understanding and management of bioeroded surfaces, especially due to the fact that bioerosion process would progressively undermine the resistance of the rock.

## Figures and Tables

**Table 1.** Statistics of the absolute distance calculated between SFM photogrammetry and DIY-TLS point cloud datasets for *x*, *y*, *z* spatial coordinates.

**Spatial Coordinate**	**Absolute Distance (m)**		

	**Mean**	**Standard Deviation**	**Maximum**
x, y, z	0.067	0.035	0.33
x	0.028	0.023	0.22
y	0.042	0.033	0.30
z	0.029	0.025	0.25
